# Knowledge of prevention of mother to child transmission of HIV among women of reproductive age group and associated factors at Mecha district, Northwest Ethiopia

**DOI:** 10.1186/s13104-020-05005-5

**Published:** 2020-03-18

**Authors:** Tewachew Muche Liyeh, Endeshaw Admasu Cherkose, Miteku Andualem Limenih, Tigist Seid Yimer, Hailemariam Demewozu Tebeje

**Affiliations:** 1 Department of Midwifery, College of Health Sciences, Debre Tabor University, Debra Tabor, Ethiopia; 2 Department of Midwifery, College of Medicine and Health Sciences, University of Gondar, Gondar, Ethiopia

**Keywords:** Knowledge, PMTCT, HIV, Women, Ethiopia

## Abstract

**Objective:**

This study was aimed to asses knowledge of prevention of mother to child transmission of HIV among reproductive age women and associated factors at Mecha district, North West Ethiopia. A community based cross sectional study was conducted among 853 reproductive age women from July 1- 30/2016 in Mecha district. By multistage sampling technique data were collected through pre-tested questionnaire. The collected data was entered in Epi Data 3.1 and analyzed with SPSS version 20. Bivariate and multivariable logistic regression model were used.

**Result:**

About 22.4% of the respondents were knowledgeable on prevention of mother to child transmission (PMTCT) of HIV. Having knowledge on PMTCT of HIV was significantly associated with urban residence (AOR = 2.486, 95% CI 1.160–5.328), education level of secondary and above (AOR = 5.445, 95% CI 2.698–10.986), those having history of antenatal care followup (AOR = 4.430, 95% CI 1.471–13.340), those with history of institutional delivery (AOR = 4.766, 95%CI 2.004–11.334), those having comprehensive knowledge on HIV/AIDS (AOR = 1.697, 95%CI 1.011–2.846), women who were knowledgeable about mother to child transmission of HIV (AOR = 2.203, 95% CI 1.37–3.54), and women who held discussions with their husband regarding HIV/AIDS, (AOR = 2.700, 95% CI 1.658–4.396).

## Introduction

Vertical transmission of Human Immunodeficiency Virus (HIV) during pregnancy, delivery and breast feeding period continues to be a major public health problem and constitutes the most important cause of HIV infection in children less than 15 years old in the world [1]. Over 90% of new infections of human immunodeficiency virus in infants and young children occur through mother-to-child transmission [2]. In Ethiopia it is estimated that 109,133 children less than 15 years were living with HIV in 2016 and there were an estimated of 2420 new infections each year due to mother-to-child transmission. The reasons for an increasing mother to child transmission (MTCT) of HIV might include lack of knowledge of mothers on prevention of mother to child transmission (PMTCT) [3].

Knowledge of reproductive age women on prevention of mother-to-child transmission (MTCT) of HIV plays a major role in limiting the number of children being infected by HIV. With timely interventions like testing for HIV during pregnancy, safe delivery practices, preventive anti-retroviral (ARV) drugs, and modified infant feeding practices the risk of a baby getting HIV infection from an infected mother can be reduced from 20–45% to 2–5% [4–6]. Therefore maternal knowledge on PMTCT is a corner stone of effective implementation of the four pronged approach to reduce mother-to-child transmission of HIV.

Even though there were some studies done at the institution level; as far as our knowledge is concerned, there is no evidence about knowledge of women on PMTCT and associated factors at the community level in Ethiopia generally and in the study area particularly.

With scarce availability and accessibility of health institution in Ethiopia, significant number of the population failed to visit the health facility. Studies conducted at institution level can’t inferred the entire population at large and community based study was so mandatory. Therefore this study was conducted to identify knowledge of reproductive age women on PMTCT of HIV and associated factors at the community level.

## Main text

### Method

#### Study setting and participants

Community based cross sectional study was conducted from July 1- 30/2016 in Mecha district, Ethiopia. The district has an estimated population size of 383,861 among whom 82,506 were reproductive age group in 2016. The source population were all reproductive age women in Mecha district and the study population was reproductive age women living in selected kebeles in Mecha district during study period.

#### Sample size determination and sampling procedure

Sample size was determined by using single population proportion formula with the assumptions of 95% level of confidence, 50% proportion, 5% of margin of error and design effect of two. Finally, considering a nonresponse rate of 10%, the total sample size was 853. Multi-stage sampling technique was used to select the study participants. There are 6 Urban and 40 rural kebeles with in the district. First kebeles were stratified into urban and rural kebeles. Then 2 urban and 8 rural kebeles were randomly selected by lottery method. Census was carried out in selected urban and rural kebeles to identify the households with reproductive age woman in the district. The sample size was distributed to each kebele proportional to the household size. Then individual households in the chosen kebeles were selected by using a systematic sampling technique. In the case of more than one eligible participant in the household, lottery method was used to select only one. For eligible participant which was not found at home, the interviewers went to the next house hold.

### Data collection tools and techniques

Data were collected by using pretested interviewer administered questionnaire. The collected data was reviewed and checked for completeness before data entry.

#### Variables

The dependent variable was knowledge on PMTCT of HIV and the independent variables were; socio-demographic characteristics, reproductive characteristics, comprehensive knowledge of HIV/AIDS and knowledge on MTCT of HIV/AIDS.

#### Operational definition

Five multiple choice questions were used to measure knowledge of PMTCT. Each correct response was given a score of 1 point and a wrong response a score of 0 point. Responses were summed, and the mean score value was calculated. Score above or equal to the mean value was categorized as knowledgeable on PMTCT and below the mean value was considered as not knowledgeable.

The women who identified correctly the three different periods of MTCT of HIV (during pregnancy, childbirth and breast feeding) considered as knowledgeable on MTCT.

#### Data analysis

The data were analyzed by using SPSS version 20. Bivariate and multivariable analysis was done for all explanatory variables and those variables with P < 0.2 were entered into multivariable logistic regression. Adjusted odds ratio with 95% confidence interval was computed and P-value less than 0.05 considered as a significant value.

#### Ethical clearance

Ethical clearance was obtained from ethical review committee of university of Gondar and permission was obtained from Mecha woreda health office. Written informed consent and assent (for participants aged less than 18 rear old) was obtained prior to data collection.

## Result

From the total of 853 reproductive age women included in the study, 830 of them responded the question correctly making the response rate of 97.3%.

### Socio demographic characteristics

The mean (± SD) age of the respondents were 28.5 ± 8.02 and 210 (25.3%) of the women were within the age group of 20–24 years. Majority 640 (77.1%) of the study population were from rural area (Table 1).

**Table 1:** Sociodemographic characteristics of respondents at Mecha district, Ethiopia, 2016

Variables	Category	frequency	Percent (%)
Age	< 20	96	11.6
	21–30	369	44.5
	31–40	247	29.7
	> 40	118	14.2
Residence	Rural	640	77.1
	Urban	190	22.9
Marital status	Married	775	93.4
	Single	42	5.1
	Divorce	6	0.7
	Widowed	7	0.8
Educational status	No formal education	686	82.7
	Primary education	34	4.1
	Secondary and above	110	13.3
Occupation	House wife	738	88.9
	Gov’t employee	36	4.3
	Market trade vender	20	2.4
	Daily laborer	28	3.4
	Student	8	1.0
Distance from health institution	0.1–5 km	711	85.7
	> 5 km	119	14.3

### Reproductive health characteristics

Concerning the reproductive status of the women, 389 (46.9%) were multipara. About 84.9%, 82.5% and 61.3% of the respondents had history of using family planning, ANC follow up and institutional delivery respectively.

### Comprehensive knowledge of the women about HIV/AIDS

Four hundred six (48.9%) of the respondents had comprehensive knowledge about HIV/AIDS. Nearly one-fifth, 19.2 and 6.5% of the respondents described that HIV can be transmitted by mosquitos and by super natural powers respectively. Most 757 (91.2%) of them knew that healthy-looking person may have AIDS virus. About 73.3% of the respondents knew that someone can prevent from HIV by consistent condom use and limiting sex partners.

### Knowledge of the women on MTCT

Six hundred sixty-one (79.6%) knew that HIV could be transmitted from an infected mother to her baby. Concerning the time of transmission of the virus from the infected mother to her child, 77.9%, 50.2% and 49.9% responded that MTCT could be through breast feeding, during delivery and during pregnancy respectively. Over all 221 (26.6%) of the respondents were knowledgeable on MTCT of HIV.

### Knowledge of the Women on PMTCT

The study assessed knowledge of women on PMTCT of HIV/AIDS.More than three-fourths (76.6%) of the respondents had heard about PMTCT of HIV of whom 186 (22.4%) of the respondents were knowledgeable on PMTCT of HIV. Three hundred thirty-six 336 (52%) of participants knew that abstinence from breast feeding could reduce mother to child transmission (MTCT) of HIV (Table 2).

**Table 2:** Kowledge of women about PMTCT, at Mecha district, Ethiopia, 2016

Variables	Category	Frequency	Percent (%)
Heard about PMTCT	Yes	636	76.6
	No	194	23.4
Time of initiation of ANC drug	First trimester	530	83.4
	Second trimester	35	4.2
	Third trimester	27	3.3
	I am not sure	44	9.1
	Total	636	100
Time of initiation of ART prophylaxis for the newborn	Immediately after delivery	501	78.8
	After 1 month	58	9.1
	After 6 month	19	3.0
	Don’t know	58	9.1
	Total	636	100
What should an HIV + mother feed her baby to prevent MTCT	Breast milk	418	65.8
	Cow milk	85	12.4
	Formula feeding	77	12.1
	I don’t know	56	9.7
	Total	636	100
Can abstinence from breast feeding could reduce MTCT	Yes	336	52
	No	300	48
	Total	636	100
Preferable mode of delivery to reduce MTCT	C/S	101	15.9
	Instrumental delivery	204	32.1
	SVD	273	42.9
	I don’t know	58	9.1
	Total	636	100
knowledge on PMTCT	Knowledgeable	186	22.4
	Non knowledgeable	644	77.6
	Total	830	100

### Factors associated with knowledge of women on PMTCT of HIV

Compared to women who live in the rural areas, those women living in the urban areas were 2.5 times (AOR = 2.486, 95% CI 1.160–5.328) more likely to be knowledgeable on PMTCT of HIV. Women with education level of secondary and above were 5.4 times (AOR = 5.445, 95% CI 2.698–10.986) more likely to be knowledgeable on PMTCT of HIV than those with no formal education.

Women who had history of ANC follow up were 4.4times (AOR = 4.430, 95% CI 1.471–13.340) more knowledgeable on PMTCT of HIV/AIDS than who hadn’t ANC follow up. Women who had history of institutional delivery were more knowledgeable about PMTCT (AOR = 4.766, 95% CI 2.004–11.334) than those who didn’t have.

Women who were knowledgeable on comprehensive knowledge on HIV/AIDS were 1.7 times (AOR = 1.697, 95% CI 1.011–2.846) more likely to be knowledgeable on PMTCT of HIV than non- knowledgeable counter parts. Women who were knowledgeable on MTCT of HIV were 2.2 times (AOR = 2.203, 95% CI 1.369–3.544) more knowledgeable on PMTCT of HIV than those who did not have.

Women who had discussions with their husband about HIV/AIDS, MTCT and its prevention were 2.7 times (AOR = 2.700, 95% CI 1.658, 4.396) more likely to be knowledgeable than those who had not (Table 3).

**Table 3:** Association between knowledge of PMTCT and explanatory variables among reproductive age women on Mecha district, Ethiopia, 2016

Variables	Category	Knowledgeable on PMTCT		COR (95% CI)	AOR (95% CI)	P-value
		Yes	no			
Residence	Rural	80	560	1	1	
	Urban	106	84	8.83 (6.10–12.79)	2.49 (1.16–5.33)	0.019
Educational status	No formal education	102	584	1	1	
	Secondary and above	70	40	0.02 (6.44–15.59)	4.45 (2.70–10.99)	0.00
History of ANC visit	Yes	154	453	10.62 (3.86–29.23)	4.43 (1.47–13.34)	0.008
	No	4	125	1	1	
History of institutional delivery	Yes	146	300	11.99 (6.36–22.61)	4.77 (2.00–11.33)	0.000
	No	11	271	1	1	
Comprehensive knowledge about HIV/AIDS	Knowledgeable	142	264	4.65 (3.20–6.74)	1.70 (1.01–2.85)	0.045
	Non Knowledgeable	44	380	1	1	
Knowledgeable on MTCT	Yes	88	133	3.45 (2.44–4.87)	2.20 (1.37–3.54)	0.001
	No	98	511	1	1	
Discussion with husband	Yes	99	200	2.78 (1.97–3.92)	2.70 (1.66–4.40)	0.000
	No	74	415	1	1	

## Discussion

In this community based cross sectional study about 22.4% of the respondents were knowledgeable on PMTCT of HIV. This finding is less than the study conducted at Gondar (83.5%) [7], Hawasa referral hospital (82.3%) [8] and Southern Nigeria (91.4%) [9]. This discrepancy might be due to the study setting and source population difference.

Those women residing in urban areas were 2.5 times (AOR = 2.5, 95% CI 1.16–5.33) more likely to be knowledgeable when compared to the rural residents. This finding is in line with studies conducted at Hawassa referral hospital, Gondar and Tanzania [7, 8, 10]. It might be due to the urban location geographical accessibility and availability of nearby health services and greater media exposure compared with rural areas.

In this study education level of secondary and above were 5.4 times (AOR = 5.4, 95% CI 2.69–10.98) more likely to be knowledgeable on PMTCT of HIV than those with no formal education. This explanation is in line with the study done in, Addis Ababa, Hawassa and Tanzania [8, 11, 12]. This could be because when the women become educated their health seeking behavior and access to information might be increased. With this regard, they might have access to print media exposure for educated one.

Women who had history of ANC follow up were about 4.4times (AOR = 4.4, 95% CI 1.47–13.34) more likely to be knowledgeable on PMTCT of HIV/AIDS than those who hadn’t ANC followup. It could be due to women who had history of ANC follow up might get the chance to learn from health professionals and this information may enhance women’s knowledge about PMTCT. This finding is consistent with the study conducted at Hawassa referral hospital [8] despite the confidence interval in our study was found to be wide.

Women who had history of institutional delivery were more knowledgeable about PMTCT (AOR = 4.77, 95% CI 2.00–11.33) than those who had not. This finding is also consistent with the study conducted at Hawassa referral hospital [8]. This might be women who had history of institutional delivery might get the chance of PMTCT service at the health institution from health professionals.

Women who had comprehensive knowledge on HIV/AIDS were 1.7 times (AOR = 1.7, 95% CI 1.01–2.85) more likely to be knowledgeable on PMTCT of HIV than non-knowledgeable counter parts. This finding was consistent with a study conducted in Gondar and Assosa, Ethiopia [7, 13]. The possible interpretation for this positive association is that those women with comprehensive knowledge on HIV may appreciate the prevention strategies of mother to child transmission of HIV.

Women who were knowledgeable on MTCT of HIV were 2.2 times (AOR = 2.20, 95% CI 1.37–3.54) more knowledgeable on PMTCT of HIV than those who did not have. This finding is in agreement with previous study done at Assosa town, Ethiopia [13]. This might be due to women with knowledgeable on MTCT of HIV might have greater understanding on prevention possibilities.

Women who had discussions with their husband about HIV/AIDS, MTCT and its prevention were 2.7 times (AOR = 2.700, 95% CI 1.7, 4.4) more likely to be knowledgeable than those who had not. This finding is inline with the study done on Mekele and Southern Ethiopia [13–15]. This might be explained due to women having discussion with their husband regarding HIV/AIDS will help to share the information and increase her level of understanding which enhances her PMTCT knowledge.

## Conclusion and recommendation

Despite many efforts invested through health extension workers, level of Knowledge on PMTCT among women was found to be low. Residence, education level ANC follow up, history of institutional delivery, comprehensive knowledge on HIV/AIDS, knowledge on MTCT of HIV and women who held discussions with their husband about HIV/AIDS were significantly associated with women’s knowledge on PMTCT. Emphasis shall be given on continuous education regarding PMTCT, strengthening ANC and institutional delivery coverage with integrated PMTCT service. Moreover, strengthening discussion of PMTCT with their spouses is quite important.

## Limitation

Being cross-sectional study might make difficult to draw conclusion about cause effect relationship.

## Data Availability

The datasets generated during the current study are available from the corresponding author on reasonable request.

## Abbreviations

AIDS: Acquired immunodeficiency syndrome
ART: Anti-retroviral therapy
MTCT: Mother to child transmission
PMTCT: Prevention of mother to child transmission
